# A pilot study of novel duodenal covered self-expandable metal stent fixation

**DOI:** 10.1038/s41598-021-99265-1

**Published:** 2021-10-05

**Authors:** Yasuki Hori, Kazuki Hayashi, Itaru Naitoh, Katsuyuki Miyabe, Makoto Natsume, Michihiro Yoshida, Hiromi Kataoka

**Affiliations:** Department of Gastroenterology and Metabolism, Nagoya City University Graduate School of Medical Sciences, 1 Kawasumi, Mizuho-cho, Mizuho-ku, Nagoya, 467-8601 Japan

**Keywords:** Gastrointestinal cancer, Gastric cancer, Pancreatic cancer, Gastroenterology

## Abstract

Migration of duodenal covered self-expandable metal stents (C-SEMSs) is the main cause of stent dysfunction in patients with malignant gastric outlet obstruction (mGOO). Because endoscopic SEMS placement is frequently selected in patients with poor performance status, we concurrently focused on the safety of the treatment. This pilot study included 15 consecutive patients with mGOO who underwent duodenal partially covered SEMS (PC-SEMS) placement with fixation using an over-the-scope-clip (OTSC). Technical feasibility, clinical success for oral intake estimated by the Gastric Outlet Obstruction Scoring System (GOOSS) score, and adverse events including stent migration were retrospectively assessed. All procedures were successful, and clinical success was achieved in 86.7% (13/15). Mean GOOSS scores were improved from 0.07 to 2.53 after the procedure (*P* < 0.001). Median survival time was 84 days, and all patients were followed up until death. Stent migration occurred in one case (6.7%) at day 17, which was successfully treated by removal of the migrated PC-SEMS using an enteroscope. For fixation using an OTSC, additional time required for the procedure was 8.9 ± 4.1 min and we did not observe OTSC-associated adverse events. Poor performance status was associated with clinical success (*P* = 0.03), but we could provide the treatment safely and reduce mGOO symptoms even in patients with poor performance status. In conclusion, duodenal PC-SEMS fixation using an OTSC is feasible for preventing stent migration in patients with mGOO including those with poor performance status.

## Introduction

The clinical syndrome of malignant gastric outlet obstruction (mGOO) occurs as a result of a narrowing in the region of the gastroduodenum. Two main etiologies for mGOO are pancreatic and gastric cancer. Several randomized controlled trials (RCTs) have evaluated endoscopic self-expandable metal stent (SEMS) placement and surgical gastrojejunostomy (GJ) for palliation of incurable mGOO^1,2^. No differences were observed in technical success, major adverse event rates, or postoperative mortality. Shorter time to resumption of oral intake and shorter recovery time with SEMS placement may decrease the time interval before palliative chemotherapy^3,4^. According to a recent published American Society for Gastrointestinal Endoscopy guideline^5^, although SEMS placement may offer short-term advantages, patients whose performance status is good and whose life expectancy is longer than 6 months may benefit more from surgical GJ than SEMS placement. A patient with poor performance status who would be unable to tolerate surgical GJ may be an appropriate candidate for endoscopic SEMS placement. Clinicians should evaluate treatment options carefully to optimize care for patients.

Endoscopic duodenal SEMS placement may be selected because it is minimally invasive. Uncovered SEMSs (U-SEMSs) and covered SEMSs (C-SEMSs) (including partially covered SEMSs [PC-SEMSs]) are available, and RCTs^6–10^ have revealed their clinical features. The main cause of stent dysfunction with U-SEMSs is tumor ingrowth via the mesh of the stent, which occurs in 16–44%^6–9^ of cases. Chemotherapy is reported as the only method to prevent tumor ingrowth^11,12^, but in actual clinical practice more than 70% of patients with mGOO are not eligible for chemotherapy due to disease progression^13^. C-SEMSs were designed to prevent tumor ingrowth, but the incidence of stent migration reportedly ranges from 6 to 32%^6–11,14–24^. Chemotherapy that results in a decrease of tumor volume and reduction of alimentary tract compression is recognized as a major predictive factor for stent migration^11^. Improvements in the shape of the stent have been attempted to prevent C-SEMS migration, but the results have been controversial and unsatisfactory.

Anchoring of C-SEMSs with devices has been proposed as another countermeasure against C-SEMS migration^25,26^. A recent systematic review^27^ has reported that OTSC was used for stent fixation in only 20 patients. Although from the limited cases, the overall technical and clinical success rate was 100.0% and 80.0%, respectively. In the systematic review, the largest paper (n = 12) was presented by Mudumbi et al*.*^28^; the major indication for endoscopic stenting with OTSC fixation was tracheo-esophageal fistula. We previously evaluated the safety and effectiveness of duodenal C-SEMS fixation using suturing and an over-the-scope-clip (OTSC) device (Ovesco Endoscopy, Tübingen, Germany) in an experimental model^26^. However, to the best of our knowledge, no clinical trial has been conducted using these methods for duodenal SEMS fixation. Endoscopic suturing device is not available for clinical use in our country; therefore, we conducted this pilot study to evaluate the technical feasibility and clinical effectiveness of duodenal PC-SEMS fixation using OTSC in patients with mGOO.

## Results

Table 1 lists the demographic and clinical characteristics of all patients. Patients included 10 males (66.7%), and the median age of all patients was 78 years (range 43–96). Pancreatic cancer (n = 9, 60.0%) and gastric cancer (n = 3, 20.0%) were the two main etiologies. Fourteen patients (93.3%) had no oral intake (GOOSS score 0), and 3 patients (20.0%) were unable to care for themselves (Group C; KPS score [0 − 40]).

**Table 1:** Patient characteristics.

Case	Age	Gender	Diagnosis	Site of obstruction	Karnofsky performance status (category^a^)	Pre-GOOSS score	Ascites	Liver metastasis	Peritoneal dissemination
1	80	M	Gastric cancer	Stomach	60 (B)	0	No	No	No
2	80	M	Pancreatic cancer	Bulb	80 (A)	0	No	Yes	No
3	96	F	Gastric cancer	Stomach	50 (B)	0	No	No	No
4	57	M	Pancreatic cancer	Second portion	20 (C)	0	Yes	Yes	Yes
5	74	M	Pancreatic cancer	Bulb	50 (B)	0	No	Yes	No
6	78	M	Gallbladder cancer	Bulb	90 (A)	0	No	Yes	No
7	78	F	Pancreatic cancer	Third portion	90 (A)	1	No	No	No
8	80	M	Pancreatic cancer	Third portion	80 (A)	0	No	No	No
9	71	M	Pancreatic cancer	Third portion	20 (C)	0	No	No	No
10	95	F	Pancreatic cancer	Third portion	50 (B)	0	Yes	No	Yes
11	60	F	Pancreatic cancer	Third portion	60 (B)	0	Yes	Yes	Yes
12	48	M	Gastric cancer	Second portion	10 (C)	0	No	Yes	Yes
13	54	M	Pancreatic cancer	Bulb	80 (A)	0	Yes	No	Yes
14	43	M	Colon cancer	Second portion	80 (A)	0	No	Yes	Yes
15	81	F	Renal cancer	Third portion	80 (A)	0	No	No	Yes

GOOSS, gastric outlet obstruction scoring system.

^a^According to the assessment by Karnofsky performance status, patients are divided into three groups: Group A (80–100) can independently perform daily activities, Group B (50–70) can perform daily activities with help, and Group C (0–40) requires continuous assistance and progressively approaches death.

### Technical and clinical outcomes

Table 2 lists treatment outcomes and adverse events. The technical success rate was 100.0% (15/15), and successful application of the OTSC for fixation was accomplished in all patients. The mean procedure time required for OTSC placement was 8.9 ± 4.1 min, and no adverse events were related to the fixation method. The total median procedure time for PC-SEMS placement with fixation was 32.1 ± 8.5 min. Clinical success was achieved in 13 patients (86.7%), and mean GOOSS scores were improved from 0.07 to 2.53 after PC-SEMS placement with fixation (*P* < 0.001). Eight patients (53.3%) were able to have full diets. The clinical success rate as estimated by the GOOSS score was significantly associated with KPS status (Groups A and B, 100.0% [12/12]; Group C, 33.3% [1/3]; *P* = 0.03). Chemotherapy was performed after SEMS placement in 4 patients (26.7%) as appropriate. All selected regimens were S-1 monotherapy.

**Table 2:** Treatment outcomes and adverse events.

Case	Technical success	Clinical success	Procedure time for OTSC placement (min)	Chemotherapy after SEMS placement, regimen	Post-GOOSS score	Adverse event (days)	Overall survival (days)
1	Yes	Yes	9	No	3	–	149
2	Yes	Yes	11	No	3	Migration, 17	35
3	Yes	Yes	21	No	2	–	134
4	Yes	No	9	No	1	–	17
5	Yes	Yes	12	No	3	–	84
6	Yes	Yes	6	Yes, S-1	3	–	98
7	Yes	Yes	12	Yes, S-1	3	–	192
8	Yes	Yes	8	No	3	–	91
9	Yes	Yes	8	No	2	–	20
10	Yes	Yes	8	No	3	–	84
11	Yes	Yes	8	No	2	–	15
12	Yes	No	4	No	1	–	23
13	Yes	Yes	9	Yes, S-1	3	–	101
14	Yes	Yes	3	Yes, S-1	3	–	74
15	Yes	Yes	5	No	3	–	115

*GOOSS* gastric outlet obstruction scoring system, *OTSC* over-the-scope-clip, *SEMS* self-expandable metal stent.

### Adverse events and follow-up

**Figure 1:**
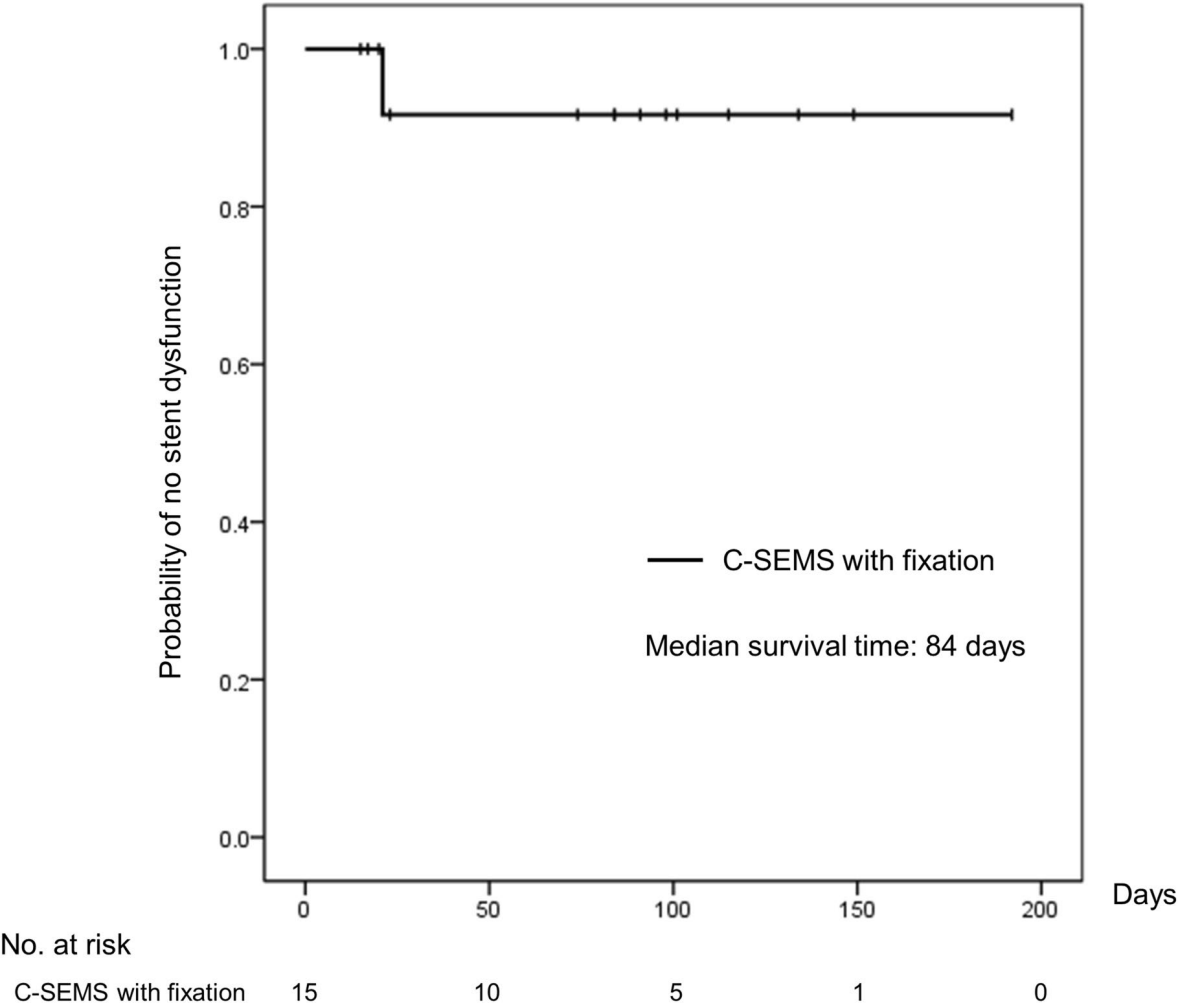
Cumulative stent patency was analyzed by using the Kaplan–Meier method. The median survival time of the study cohort was 84 days.

One case (6.7%) had stent dysfunction related to PC-SEMS migration at day 17, which was successfully treated by removal of the migrated PC-SEMS using an enteroscope. No other adverse events, including perforation and tumor overgrowth, were observed during the remainder of the patients’ lives. Median survival time was 84 days (range 15–192) and all patients were followed up until death. All patients died of their underlying carcinoma. Figure 1 presents the cumulative stent patency curve estimated by Kaplan–Meier analysis.

## Discussion

These results demonstrated that duodenal PC-SEMS placement and fixation with an OTSC for mGOO was successful in all cases and that 86.7% of the patients achieved clinical success. In particular, 53.3% of patients with mGOO could have full diets. The additional time required for the procedure was 8.9 ± 4.1 min, which may be within the permissible range. Moreover, no adverse events were related to the fixation method. Poor performance status was associated with clinical success as estimated by the GOOSS score (*P* = 0.03). Stent migration occurred in one case (6.7%) with no other adverse events. No asymptomatic stent migration was observed on periodic abdominal X-ray imaging.

Stent migration is a major adverse event of duodenal C-SEMSs. Kim et al*.*^6^ reported that stent migration was significantly associated with chemotherapy after stent placement. Isayama et al*.*^17^ recommended the use of longer stents to prevent stent occlusion caused by tumor in- or overgrowth at the uncovered portion. According to this recommendation, in this pilot study, we used the longest PC-SEMS (120 mm) available in our country. We were fortunate to experience no cases of tumor in- or overgrowth. Once a duodenal C-SEMS has migrated into the jejunum, clinicians hope it will exit via the rectum or remain in the body without causing obstruction symptoms. Otherwise, with obstruction symptoms, surgical removal is required, and this exhausts patients with advanced cancer. In this study, we experienced one case with stent migration. The patient (case no.2) did not receive chemotherapy. Seventeen days after the procedure, patient was admitted to the emergency ward with abdominal distension and vomiting. As the position of migrated metal stent did not change, we decided to remove it using enteroscope (day 19). But the enteroscope could not pass the duodenal stenosis, we placed a second duodenal SEMS. The second SEMS was fully expanded at day 21, and we could pass the duodenal stenosis. Fortunately, we were able to remove the migrated PC-SEMS using an enteroscope without surgery^29^. It is important to accommodate asymptomatic migration, which is not included in stent dysfunction. Although this event fortuitously may not cause symptoms related to gastrointestinal obstruction, it potentially results in intestinal obstruction.

Table 3 lists some published articles about endoscopic duodenal covered metal stenting for mGOO^6–11,14–24^. The literature review assessed more than 1000 cases of endoscopic duodenal C-SEMS stenting and found that chemotherapy was administered after C-SEMS placement in 41.2% (range 11.4–78.6%), and that stent migration occurred in 14.5% (range 6.0–32.3%) of cases. Not all of the clinical studies carried out periodic abdominal X-ray imaging or follow-up endoscopy, so the real rate of stent migration might be higher because asymptomatic stent migration could have been missed. Kim et al*.*^6^ performed a routine 8-week follow-up endoscopy and found that 63% of cases with a distally migrating C-SEMS occurred in patients without any obstructive symptoms. Their prospective study confirmed stent migration in almost one-third of patients during total follow-up. Another important issue is that the rate of patients receiving chemotherapy varies in published articles. The chemotherapy regimen continues to change and evolve, and some prospective studies including RCTs excluded mGOO patients with poor performance status^10,17^. Patients with poor performance status tend to avoid receiving chemotherapy, so the rate of patients with mGOO receiving chemotherapy might be lower in real-world practice. We did not exclude patients with poor performance status, so 26.7% of patients in our study cohort received chemotherapy after the procedure. We found that poor performance status was associated with clinical success, defined as relief of GOOSS score ≥ 2 within 1 week. Although the result could be due to underlying disease, symptoms related to mGOO were relieved in all cases (at least 1 GOOSS score improvement). Endoscopic duodenal PC-SEMS placement with fixation might be beneficial even in patients with poor performance status.

**Table 3:** Migration rate of published endoscopic gastroduodenal covered stenting.

Author and reference	Year	Study type	Number of patients (C-SEMS only)	Stent type	Chemotherapy after C-SEMS placement (%)	Migration rate (%)	Median survival time or follow-up duration (days)
Bang et al*.*^14^	2008	Retrospective	53	Niti-S pyloric stent	NA	26.4	121
Lee et al*.*^15^	2009	Consecutive	70	Niti-S pyloric stent	11.4	17.1	115
Maetani et al*.*^16^	2009	Retrospective	29	Ultraflex esophageal	20.7	6.7	62
Kim et al*.*^6^	2010	RCT	40	Niti-S pyloric stent and ComVi stent	67.5	32.3	101.5
Isayama et al*.*^17^	2012	Consecutive	50	Modified ComVi stent	NA	6.0	106
Park et al*.*^18^	2013	Retrospective	96	Niti-S pyloric stent and ComVi stent	63.5	23.0	84
Woo et al*.*^19^	2013	Retrospective	24	Niti-S enteral and BONASTENT	20.8	20.8	63
Kim et al*.*^20^	2014	Retrospective	29	Niti-S pyloric stent and ComVi stent	17.2	20.7	60
Lim et al*.*^7^	2014	RCT	59	ComVi stent	39.0	13.6	113
Maetani et al*.*^8^	2014	RCT	31	ComVi stent	29.0	6.5	73
Lee et al*.*^9^	2015	RCT	42	WAVE-covered SEMS	78.6	9.5	112
Jung et al*.*^21^	2016	Retrospective	93	NA	NA	14.0	NA
Hori et al*.*^11^	2017	Retrospective	126	Ultraflex esophageal and ComVi stent	38.1	8.7	86
Takahara et al*.*^22^	2017	Retrospective	41	Flared-ComVi stent	53.7	23.1	176
Choi et al*.*^23^	2018	Retrospective	63	BONASTENT WING	58.7	11.1	176
Choe et al*.*^24^	2018	Retrospective	24	HANAROSTENT Pylorus/duodenum Kim’s Flare	12.5	16.7	99
Yamao et al*.*^10^	2020	RCT	182	Flared-ComVi stent	36.3	12.1	NA
All clinical trials (range)			1052		41.2 (11.4–78.6)	14.5 (6.0–32.3)	
Hori et al*.*, current study	2021	Consecutive	15	Flared-ComVi stent with OTSC fixation	26.7	6.7	84

*C-SEMS* covered self-expandable metal stent, *NA* not available (or no details), *OTSC* over-the-scope-clip, *RCT* randomized controlled trial.

**Figure 2:**
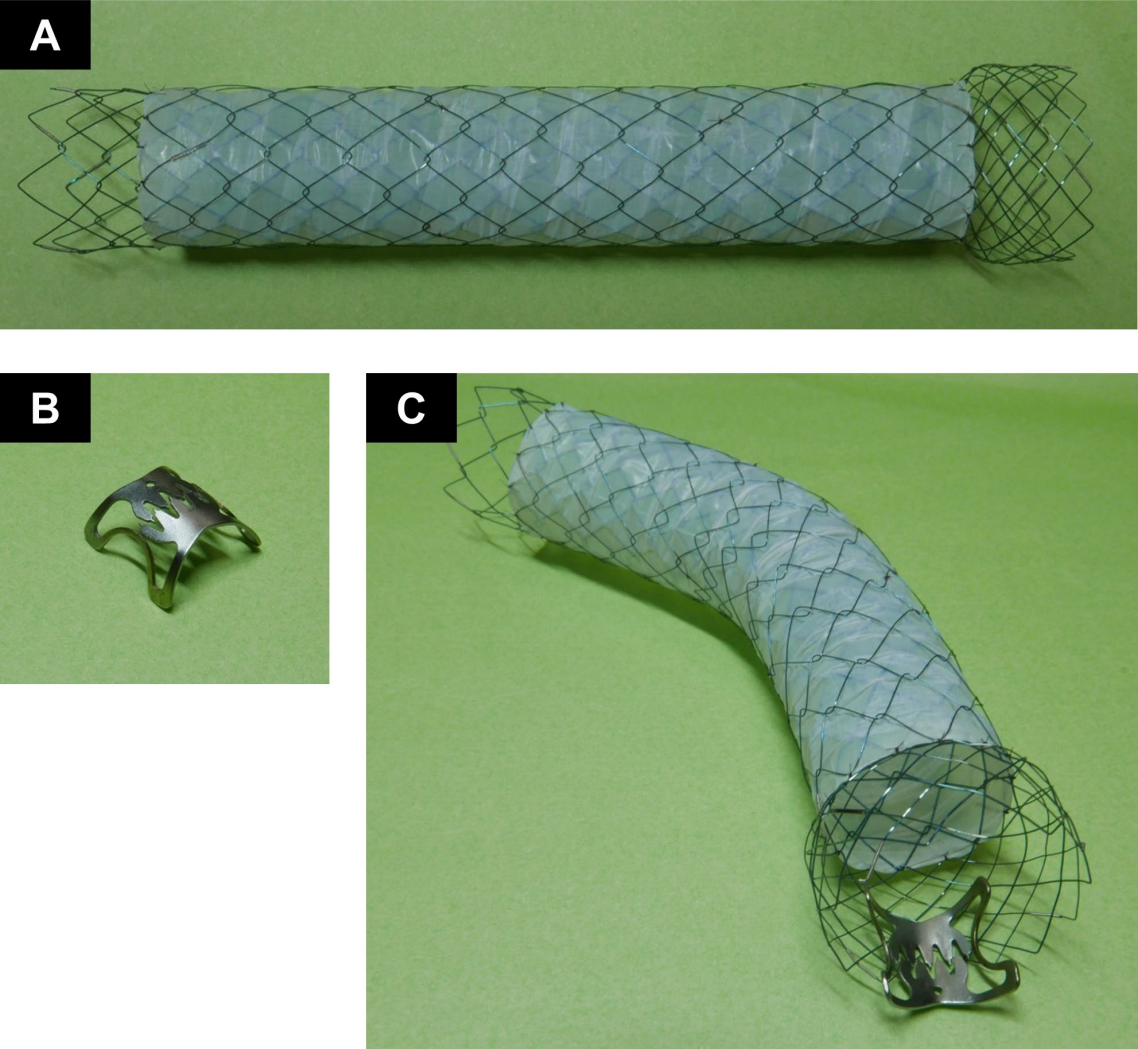
Devices and ex vivo image of this study. (**A**) The partially covered self-expandable metal stent (PC-SEMS) is 20 mm in diameter and 120 mm in length, with an uncovered flare (15 mm in length) at both ends. The proximal flare was 25 mm in diameter. (**B**) Over-the-scope-clip (OTSC). (**C**) Ex vivo image of duodenal PC-SEMS fixation. An OTSC is attached to the proximal flare of the PC-SEMS placed in the gastrointestinal obstruction.

**Figure 3:**
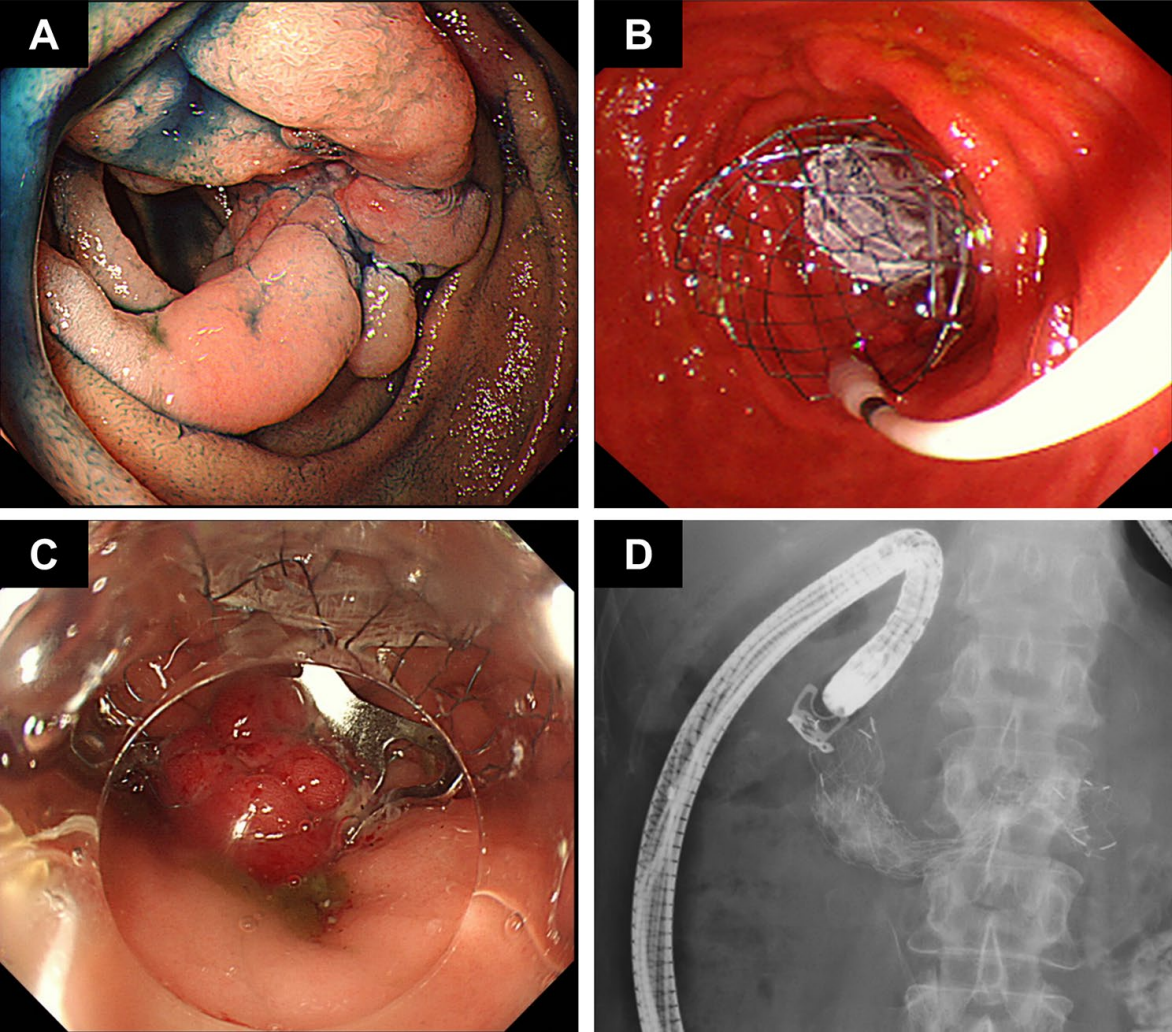
A 60-year-old female with pancreatic cancer. The third portion of the duodenum was obstructed by the tumor (**A**), and a partially covered self-expandable metal stent (PC-SEMS) was deployed (**B**). The over-the-scope-clip (OTSC) system was loaded onto the endoscope, and part of the upper rim of the metal stent was suctioned into the transparent cap. The OTSC was released to grasp both the metal stent and duodenal wall (**C**). A fluoroscopic image after OTSC and PC-SEMS placement (**D**).

Previously, because there were no designated duodenal C-SEMSs, clinicians applied esophageal C-SEMSs for relief of mGOO symptoms^16^. Due to the bended anatomy of the duodenum, stent fracture and gastrointestinal perforation became a serious problem. Recently, SEMSs with low axial force^30^ (i.e., those that follow the gastrointestinal tract) have been preferred and widely used. As shown in Figs. 2C and 3D, a SEMS with low axial force resulting from the devised knit pattern easily follows the gastrointestinal tract. Both ends of the uncovered portion were designed to stick to the tumor to prevent migration. Furthermore, the range of proximal flare was widened (Fig. 2A). Even with these ingenious improvements, previous studies using the PC-SEMS (flared-ComVi stent)^10,22^ could not prevent stent migration with complete satisfaction. In comparison, although our pilot study included a limited number of cases, we observed favorable results (23.1% and 12.1% [flared-ComVi stent only] vs 6.7% [flared-ComVi stent with OTSC fixation]). In previous attempts to prevent stent migration, Choi et al*.*^23^ used a C-SEMS with large star-shaped flaps and Choe et al*.*^24^ used a 40-mm diameter funnel shape. Those stents could prevent distal migration (no cases in both studies), but proximal migration occurred in 11.1% and 16.7% of cases, respectively. Thus, stent migration is still a challenging problem, but our fixation method that anchors C-SEMSs to the gastrointestinal tract may contribute to approaching the ideal endoscopic SEMS placement.

Not enough is known about the role of anchoring of duodenal C-SEMSs in preventing migration. One previous study^25^ reported the usefulness of endoscopic clips generally used for closing perforations or controlling bleeding to prevent C-SEMS migration. We previously practiced this technique, but still encountered cases of C-SEMS migration, and even without stent migration the clips were often out of place as assessed by routinely performed abdominal X-rays. Therefore, we evaluated the feasibility and safety of anchoring methods using experimental models^26^. The OTSC and suturing system had a significantly higher gripping force compared with the clipping system (OTSC vs. clip: 13.2 vs. 1.0 Newtons [N], *P* < 0.001; suture vs. clip: 8.5 vs. 1.0 N, *P* < 0.001). Based on pathological findings in a porcine model, OTSC compressed the submucosal layer but not the muscle layer, and we concluded that the method may be safe even for preventive use. The results revealed that SEMS fixation with an OTSC and suturing method is feasible compared with the clipping method. Because an endoscopic suturing device is not available for daily clinical use in our country, we conducted duodenal PC-SEMS fixation using OTSC in patients with mGOO. In this pilot study, we did not observe adverse events, such as gastrointestinal perforation, related to OTSC placement. We also confirmed that the position of PC-SEMSs and OTSCs was unchanged until the last follow-up in 93.3% of cases. Our technical point of the OTSC application is that we suction edge of the placed SEMS at the center part of the OTSC system. We recommend to grab normal mucosa at one side of the OTSC with enough suction. Further large-scale research using our fixation method will be required to establish higher quality evidence. The one problem with this fixation method is the additional cost (79,800 Japanese yen; approximately 770 US dollars) for an OTSC. Further studies should be planned considering medical costs.

This study had some limitations. First, it was a pilot study with a limited number of cases, and was conducted at a single institution. Further large-scale multicenter prospective research will be required to verify the superiority of our method over other conventional treatments and assess medical costs. Second, we selected an OTSC for fixation to anchor the PC-SEMS; other methods such as an endoscopic suturing system should be evaluated. Furthermore, a novel specific clip fixation device (stentfix OTSC, Ovesco Endoscopy AG, Tübingen, Germany) has been introduced and reported^31^. Currently, these system and device are not available in our country; we hope it will be accepted as a daily clinical procedure in the future.

In conclusion, an OTSC used for fixation of duodenal PC-SEMSs was safe and feasible for preventing stent migration in patients with mGOO. The treatment may bring benefits even for patients with poor performance status. We believe our efforts will contribute to establishing an anti-migratory method for duodenal PC-SEMSs.

## Methods

### Patients

Eighteen patients with mGOO were assessed for eligibility at Nagoya City University Graduate School of Medical Sciences between October 2018 and April 2020. Three patients were declined to participate in the study. In total, 15 patients underwent duodenal PC-SEMS placement and fixation with an OTSC for mGOO (Supplementary Fig. 1). Procedural and clinical data were collected and analyzed retrospectively from a prospectively maintained endoscopy database. The inclusion criteria were obstruction of the stomach or duodenum causing nausea, vomiting that reduced oral intake, or weight loss; and unresectable malignant pyloroduodenal obstruction, as shown by endoscopic or radiographic findings. The exclusion criteria were multiple gastrointestinal tract stenoses; previous treatment of GOO or transpapillary biliary stricture; and refusal to participate in the study. All patients provided written informed consent before the procedure in accordance with the Helsinki Declaration, and the study was approved by the Institutional Review Board of the Nagoya City University Graduate School of Medical Sciences (approval no. 46-18-0009) (clinical trial registration number: UMIN000034510, date of registration: 15/10/2018).

### Devices

All PC-SEMSs used in this study were flared-ComVi stents (Taewoong Medical, Seoul, Korea), 20 mm in diameter and 120 mm long. The stents had an uncovered flare (15 mm long) at both ends. The proximal uncovered flare was 25 mm in diameter and was expected to prevent stent migration (Fig. 2A). The type of OTSC was 12/6 t, which has an OTSC cap diameter of 17.5 mm and clip width of 10 mm (Fig. 2B). The OTSC clips are offered in three different shapes (atraumatic [a], traumatic [t], and gastric closure [gc]). We used type ‘t’ clips that have small spikes and blunted edges allowing for both compression and anchoring into tissues. Figure 2C presents an ex vivo image of PC-SEMS placement with fixation using an OTSC.

### Stent placement and fixation technique with an OTSC

All procedures were performed with patients under deep sedation using midazolam (5–10 mg) and pethidine hydrochloride (17.5–35 mg). Duodenal stent placement was performed using a direct-viewing scope (CF-HQ290ZI; Olympus Medical Systems, Tokyo, Japan) or a side-viewing duodenoscope (TJF260V; Olympus Medical Systems, Tokyo, Japan). The endoscope was first positioned close to the gastric or duodenal stenosis site, and the GOO was evaluated endoscopically (Fig. 3A). Contrast medium was injected under fluoroscopic guidance to identify the site and length of the obstruction. The obstruction was negotiated using a 0.025-inch standard biliary guidewire and an endoscopic retrograde cholangiopancreatography (ERCP) catheter. After confirming the position of the stenosis with the ERCP catheter, we deployed the PC-SEMS and placed it under endoscopic and fluoroscopic guidance (Fig. 3B).

Subsequently, the OTSC system for fixation was loaded onto the scope (PCF-Q260AI; Olympus Medical Systems, Tokyo, Japan), and part of the upper rim of the SEMS was suctioned into the transparent cap before releasing the OTSC, grasping both the SEMS and the gastric or duodenal wall. One OTSC was placed per patient (Fig. 3C,D).

### Data analysis and follow-up

The baseline information collected included age, gender, diagnosis, site of obstruction, Karnofsky performance status (KPS) score^32^, Gastric Outlet Obstruction Scoring System (GOOSS)^33^ score, presence of ascites/liver metastasis/peritoneal dissemination, and chemotherapy after SEMS placement. The KPS is an assessment administered by a healthcare provider to assign a patient to one of 11 categories (ranging from 0 [dead] to 100 [normal activity, no evidence of disease]). According to the assessment results, patients are divided into three groups; Group A (80–100) can independently perform daily activities, Group B (50–70) can perform daily activities with help, and Group C (0–40) requires continuous assistance and progressively approaches death. The GOOSS is a scoring system to classify a patient’s level of oral intake as follows: 0, no oral intake; 1, liquids only; 2, soft solids; 3, low-residue or full diet^33^. The presence of ascites/liver metastasis/peritoneal dissemination was evaluated by computed tomography before the procedure.

The primary endpoint was technical success. The secondary endpoints were clinical success, changes in GOOSS score from before SEMS placement to 1 week after SEMS placement, and adverse events including SEMS migration. Technical success was defined as satisfactory SEMS placement and precise positioning at the obstruction site, and ability to deploy the OTSC for SEMS fixation. Clinical success was defined as a GOOSS score of ≥ 2 and relief of GOO symptoms within 1 week after SEMS insertion. Procedure time for OTSC placement was calculated from inserting the endoscope that loaded the OTSC to endoscope removal after OTSC placement. Stent migration was defined as movement out of the stricture, which was diagnosed on endoscopy and radiography. We routinely perform abdominal X-rays on days 1, 3, 7, and 14 and every 2 weeks after the procedure to detect symptomatic and asymptomatic stent migration until the patient’s death.

### Statistical analysis

Values are reported as medians with ranges. Categorical data were compared using Fisher’s exact test. Procedure time and changes in GOOSS scores are expressed as mean (± standard deviation) and were analyzed using Wilcoxon’s signed rank test. Differences were considered to be significant at *P* < 0.05. The cumulative time to stent dysfunction was evaluated using Kaplan–Meier analysis. All statistical analyses were performed using SPSS software (version 19; IBM Corporation, USA).

## Supplementary Information

Supplementary Video 1.

Supplementary Information.

Supplementary Figure 1.

Supplementary Legends.

## Abbreviations

C-SEMS: Covered self-expandable metal stent
ERCP: Endoscopic retrograde cholangiopancreatography
GJ: Gastrojejunostomy
GOOSS: Gastric Outlet Obstruction Scoring System
KPS: Karnofsky performance status
mGOO: Malignant gastric outlet obstruction
OTSC: Over-the-scope-clip
PC-SEMS: Partially covered self-expandable metal stent
RCT: Randomized controlled trial
SEMS: Self-expandable metal stent
U-SEMS: Uncovered self-expandable metal stent

## References

[1] Jeurnink SM (2010). Surgical gastrojejunostomy or endoscopic stent placement for the palliation of malignant gastric outlet obstruction (SUSTENT study): A multicenter randomized trial. Gastrointest. Endosc..

[2] Mehta S (2006). Prospective randomized trial of laparoscopic gastrojejunostomy versus duodenal stenting for malignant gastric outflow obstruction. Surg. Endosc..

[3] Kim CG (2012). Effect of chemotherapy on the outcome of self-expandable metallic stents in gastric cancer patients with malignant outlet obstruction. Endoscopy.

[4] Kobayashi S (2016). Duodenal stenting followed by systemic chemotherapy for patients with pancreatic cancer and gastric outlet obstruction. Pancreatology.

[5] Jue TL (2021). ASGE guideline on the role of endoscopy in the management of benign and malignant gastroduodenal obstruction. Gastrointest. Endosc..

[6] Kim CG (2010). Covered versus uncovered self-expandable metallic stents for palliation of malignant pyloric obstruction in gastric cancer patients: A randomized, prospective study. Gastrointest. Endosc..

[7] Lim SG (2014). Conformable covered versus uncovered self-expandable metallic stents for palliation of malignant gastroduodenal obstruction: A randomized prospective study. Dig. Liver Dis..

[8] Maetani I (2014). Placement of a triple-layered covered versus uncovered metallic stent for palliation of malignant gastric outlet obstruction: A multicenter randomized trial. Dig. Endosc..

[9] Lee H (2015). Covered metallic stents with an anti-migration design vs. uncovered stents for the palliation of malignant gastric outlet obstruction: A multicenter, randomized trial. Am. J. Gastroenterol..

[10] Yamao K (2020). Endoscopic placement of covered versus uncovered self-expandable metal stents for palliation of malignant gastric outlet obstruction. Gut.

[11] Hori Y (2017). Predictors of stent dysfunction after self-expandable metal stent placement for malignant gastric outlet obstruction: Tumor ingrowth in uncovered stents and migration of covered stents. Surg. Endosc..

[12] Hori Y (2017). Predictors of outcomes in patients undergoing covered and uncovered self-expandable metal stent placement for malignant gastric outlet obstruction: A multicenter study. Gastrointest. Endosc..

[13] Hori Y (2018). The utility and efficacy of self-expandable metal stents for treating malignant gastric outlet obstructions in patients under best supportive care. Support Care Cancer.

[14] Bang S (2008). Effectiveness of self-expanding metal stents for malignant antropyloric and duodenal obstruction with a comparison between covered and uncovered stents. Hepatogastroenterology.

[15] Lee KM (2009). Palliative treatment of malignant gastroduodenal obstruction with metallic stent: Prospective comparison of covered and uncovered stents. Scand. J. Gastroenterol..

[16] Maetani I (2009). Metallic stents for gastric outlet obstruction: Reintervention rate is lower with uncovered versus covered stents, despite similar outcomes. Gastrointest. Endosc..

[17] Isayama H (2012). Management of malignant gastric outlet obstruction with a modified triple-layer covered metal stent. Gastrointest. Endosc..

[18] Park CI (2013). What is the ideal stent as initial intervention for malignant gastric outlet obstruction?. Dig. Liver Dis..

[19] Woo SM (2013). Comparison of uncovered and covered stents for the treatment of malignant duodenal obstruction caused by pancreaticobiliary cancer. Surg. Endosc..

[20] Kim JW (2015). Comparison between uncovered and covered self-expandable metal stent placement in malignant duodenal obstruction. World J. Gastroenterol..

[21] Jung K (2016). Outcomes of endoscopically inserted self-expandable metal stents in malignancy according to the type of stent and the site of obstruction. Surg. Endosc..

[22] Takahara N (2017). A novel partially covered self-expandable metallic stent with proximal flare in patients with malignant gastric outlet obstruction. Gut Liver.

[23] Choi YK (2018). Winged partially covered self-expandable metal stent to prevent distal migration in malignant gastric outlet obstruction. Dig. Dis. Sci..

[24] Choe JW (2018). Comparison on the efficacy between partially covered self-expandable metal stent with funnel-shaped enlarged head versus uncovered self-expandable metal stent for palliation of gastric outlet obstruction. Gastroenterol. Res. Pract..

[25] Kim ID (2010). Prevention of covered enteral stent migration in patients with malignant gastric outlet obstruction: A pilot study of anchoring with endoscopic clips. Scand. J. Gastroenterol..

[26] Hori Y (2019). Feasibility and safety of duodenal covered self-expandable metallic stent fixation: An experimental study. Surg. Endosc..

[27] Bartell N (2020). Clinical efficacy of the over-the-scope clip device: A systematic review. World J. Gastroenterol..

[28] Mudumbi S (2014). Anchoring of self-expanding metal stents using the over-the-scope clip, and a technique for subsequent removal. Endoscopy.

[29] Hori Y (2019). Successful peroral endoscopic removal of migrated metal stent. Endoscopy.

[30] Isayama H (2009). Measurement of radial and axial forces of biliary self-expandable metallic stents. Gastrointest. Endosc..

[31] Zimmer V (2020). Gastrointestinal: Utilization of a novel dedicated stent fixation clip device ("stentfix OTSC") for an umbrella-type cardia stent. J. Gastroenterol. Hepatol..

[32] Mor V (1984). The Karnofsky Performance Status Scale. An examination of its reliability and validity in a research setting. Cancer.

[33] Adler DG, Baron TH (2002). Endoscopic palliation of malignant gastric outlet obstruction using self-expanding metal stents: Experience in 36 patients. Am. J. Gastroenterol..

