# Supplementary figures and images for: A pilot study of novel duodenal covered self-expandable metal stent fixation
Source: Sci Rep. 2021 Oct 5;11:19708. doi: 10.1038/s41598-021-99265-1 (PMC8492690; doi:10.1038/s41598-021-99265-1)

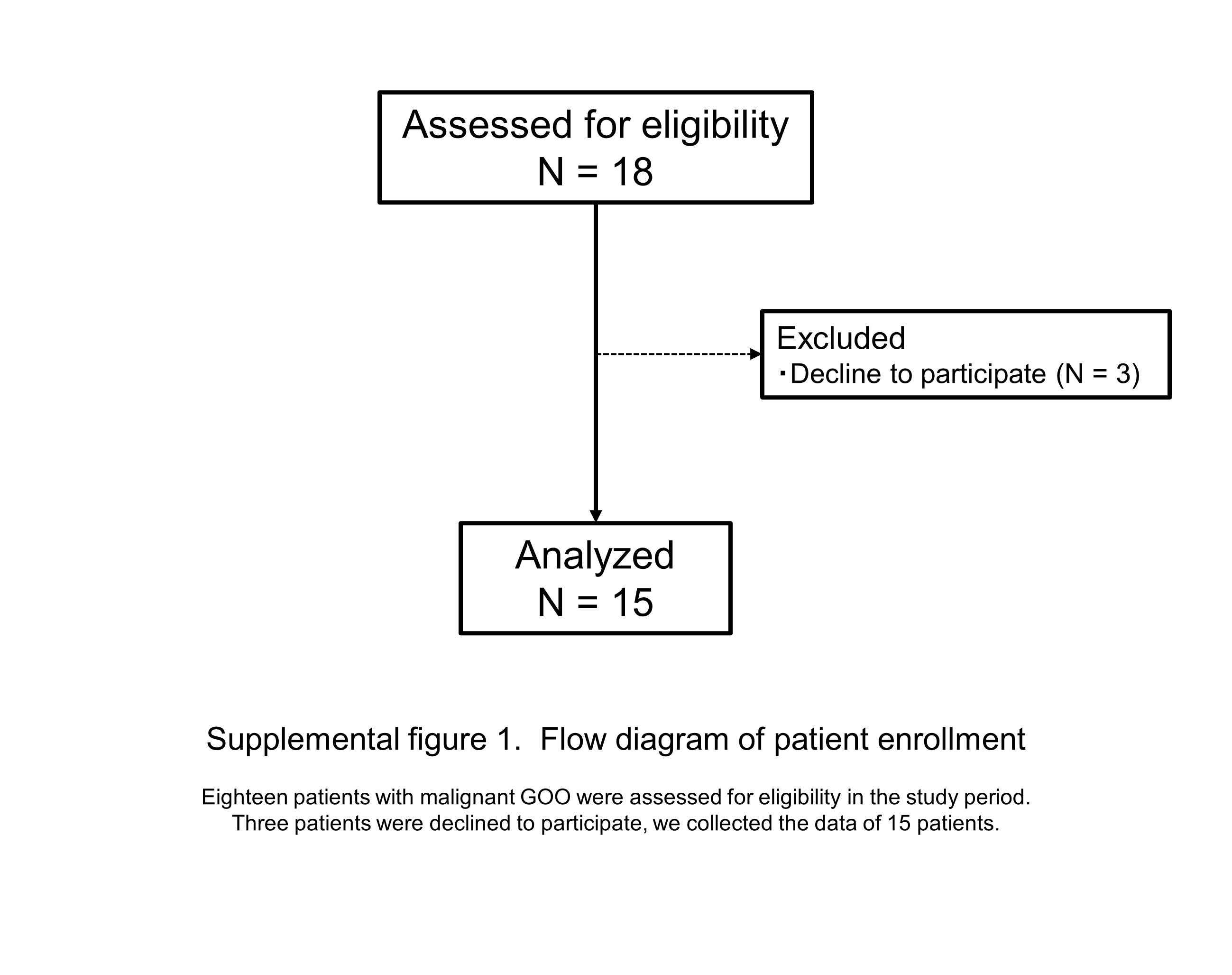

Supplement: Supplementary file 3 — Supplementary Figure 1. [file 41598_2021_99265_MOESM3_ESM.tif]
