# Supplementary material for: A pilot study of novel duodenal covered self-expandable metal stent fixation
Source: Sci Rep. 2021 Oct 5;11:19708. doi: 10.1038/s41598-021-99265-1 (PMC8492690; doi:10.1038/s41598-021-99265-1)
Supplement: Supplementary file 4 — Supplementary Legends. [file 41598_2021_99265_MOESM4_ESM.docx]

**VIDEO LEGEND**

**Video.** Endoscopic duodenal covered stent fixation

A patient was admitted to our department with malignant GOO. We placed a duodenal covered SEMS under endoscopic and fluoroscopic guidance. Subsequently, the OTSC system for fixation was loaded onto the scope. We inserted the scope with OTSC system, and the upper rim of SEMS was suctioned into the transparent cap. Grasping both the SEMS and gastric wall, and released the OTSC. Duodenal covered SEMS placement with fixation using an OTSC.
